# Diverse influences on tau aggregation and implications for disease progression

**DOI:** 10.1101/gad.352551.124

**Published:** 2025-05-01

**Authors:** Meaghan Van Alstyne, James Pratt, Roy Parker

**Affiliations:** 1 Department of Biochemistry, University of Colorado Boulder, Boulder, Colorado 80301, USA;; 2 Howard Hughes Medical Institute, University of Colorado Boulder, Boulder, Colorado 80301, USA;; 3 BioFrontiers Institute, University of Colorado Boulder, Boulder, Colorado 80301, USA

**Keywords:** neurodegeneration, protein aggregation, tau, tauopathy

## Abstract

Tau is an intrinsically disordered protein that accumulates in fibrillar aggregates in neurodegenerative diseases. The misfolding of tau can be understood as an equilibrium between different states and their propensity to form higher-order fibers, which is affected by several factors. First, modulation of the biochemical state of tau due to ionic conditions, post-translational modifications, cofactors, and interacting molecules or assemblies can affect the formation and structure of tau fibrils. Second, cellular processes impact tau aggregation through modulating stability, clearance, disaggregation, and transport. Third, through interactions with glial cells, the neuronal microenvironment can affect intraneuronal conditions with impacts on tau fibrilization and toxicity. Importantly, tau fibrils propagate through the brain via a “prion-like” manner, contributing to disease progression. This review highlights the biochemical and cellular pathways that modulate tau aggregation and discusses implications for pathobiology and tau-directed therapeutic approaches.

## Abstract (precis)

In this review, Van Alstyne et al. discuss the various molecular, biochemical, structural, and cellular features of tau. They further delineate the pathogenesis of tau mutations and the mechanisms of tau aggregation and transmission in neurological tauopathies and highlight advances in tau targeted therapeutic avenues.

Several observations support that the misfolding of tau into fibrillar aggregates is causative in >25 different neurodegenerative diseases, termed tauopathies. This was foreshadowed by the discovery that tau is the key protein in neurofibrillary tangles (NFTs), the second pathological hallmark of Alzheimer's disease (AD) (Brion et al. 1985). Critically, identification of mutations in the tau-encoding gene (*MAPT*) in frontotemporal dementia (FTD) provided the first genetic link supporting a causal role for tau in disease (Hutton et al. 1998; Poorkaj et al. 1998; Spillantini et al. 1998). Since then, >50 genetic mutations and variants in *MAPT* have been associated with familial forms of FTD, including Pick's disease (PiD), progressive supranuclear palsy (PSP), and corticobasal degeneration (CBD) (Forrest et al. 2018; Strang et al. 2019). Expression of human *MAPT* with pathogenic mutations in mice and rats also recapitulates aspects of human disease (Dujardin et al. 2015). Moreover, pathogenic variants of tau can be more prone to forming fibers in vitro, in cell lines, and in mouse models, emphasizing the importance of tau fibrillization in disease (Yoshiyama et al. 2007; Holmes et al. 2014; Chen et al. 2023a).

Tau aggregates identified in disease are composed of filaments of tau organized in β-sheet fibrillar structures (Crowther 1991; Fitzpatrick et al. 2017). Tau fibers can develop within a given cell in a “prion-like” manner, where small “seeds” of a fiber can template the misfolding of additional tau monomers into the same structure. In support of this mechanism, tau fibers are able to induce folding of naïve tau monomers into fibrils in solution (Dinkel et al. 2015; Fichou et al. 2018). Furthermore, tau fibrils can also propagate between cells, as injection of tau seeds into the mouse brain can induce aggregation across connected brain regions, reflecting the patterning observed in human pathologies (Clavaguera et al. 2009; Guo and Lee 2011; Iba et al. 2013). Another shared characteristic of prions and tau is that distinct strains defined by morphology can be stably propagated through multiple rounds of transmission between both cellular and mouse models (Sanders et al. 2014; Kaufman et al. 2016). Thus, the “prion-like” spread of tau pathology is an important aspect of disease.

In this review, we focus on the biochemical and cellular mechanisms influencing the transition of tau from a normal state into a misfolded and aggregation-prone toxic form. We also review the broader roles of both intercellular spread and contributions of the neuronal microenvironment to disease mechanisms. Last, we discuss the implications of the understanding of tau disease etiology for tau-directed therapeutic approaches. Given the breadth of the field, we apologize to those whose work we were not able to include due to space constraints.

## Biology of tau

### Tau isoforms, domain architecture, and expression

**Figure 1.**
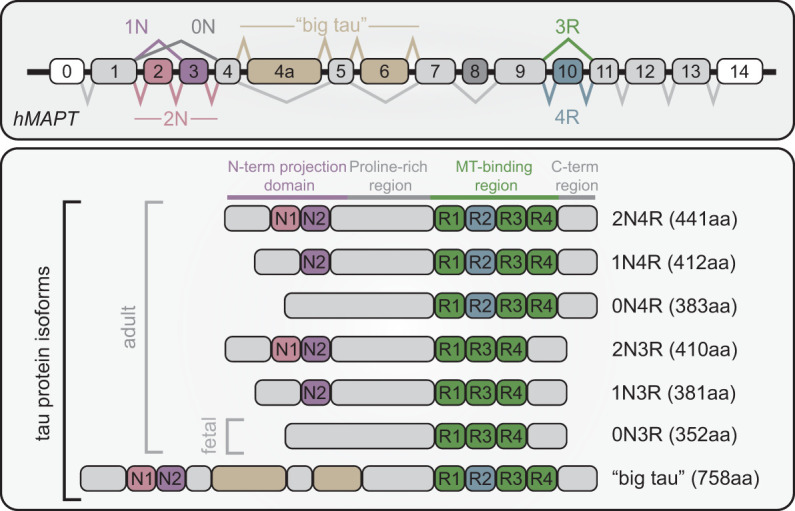
MAPT gene and tau protein isoforms. Transcripts derived from the human MAPT gene (*hMAPT*) can undergo alternative splicing to generate various tau protein isoforms. Inclusion or exclusion of exons 2 and 3 determines 0N, 1N, or 2N isoforms, whereas exon 10 determines 3R or 4R isoforms. Big tau is characterized by inclusion of exons 4a and 6. All protein isoforms are expressed in the adult human brain, whereas 0N3R is the predominant fetal isoform.

Tau is predominantly expressed in neurons, with seven alternatively spliced isoforms (Fig. 1). These isoforms are defined by the inclusion of zero, one, or two N-terminal inserts (0N, 1N, or 2N) and either three or four pseudorepeats (3R or 4R) in the microtubule binding region (MTBR) (Goedert et al. 1989a,b). Alternative splicing controls the inclusion of exons 2, 3 (0N, 1N, or 2N) and 10 (3R vs. 4R) (Andreadis et al. 1992). In embryonic and early developmental stages, tau is predominately expressed as a 3R isoform, whereas in adult brains there is a 50:50 balance of 3R:4R tau (Goedert et al. 1989a). A larger tau isoform, referred to as big tau, includes additional exons (4A and 6) in the N-terminal region and is expressed more highly in less vulnerable brain regions. (Oblinger et al. 1991; Chung et al. 2024).

Tau has four distinct domains: an N-terminal projection domain, a proline-rich region (PRR), the microtubule binding region (MTBR), and a C-terminal region. The N-terminal projection domain has no affinity for microtubules and orients away from the microtubule surface (Hirokawa et al. 1988). The PRR upstream of the MTBR has been reported to drive phase separation of tau in vitro and in cells (Zhang et al. 2020b). The PRR is also highly post-translationally modified and contains a majority of phosphorylation sites on tau (Martin et al. 2013b). The MTBR of tau is composed of three (in 3R tau) or four (in 4R tau) imperfect repeats of 30–31 amino acids that differentially mediate roles in microtubule polymerization and stability (Panda et al. 2003).

Tau is highly expressed in the brain, most predominantly in neurons, where it localizes to axons (Binder et al. 1985). The cellular spatial organization of tau is regulated by selective transport as well as localized translation and degradation (Aronov et al. 2002; Nakata and Hirokawa 2003). A change in the cellular localization of tau is part of the pathogenic process, as the mislocalization and aggregation of tau in the somatodendritic space of neurons are observed in disease (Braak et al. 1994). In addition to neurons, tau can also be expressed to a lesser degree in other central nervous system (CNS) cell types such as oligodendrocytes and astrocytes (Ezerskiy et al. 2022; Torii et al. 2023).

### Cellular roles of tau

Tau was first described as a microtubule-associated protein (MAP) that promoted microtubule polymerization in vitro (Weingarten et al. 1975; Cleveland et al. 1977; Panda et al. 1995). In cells, tau also increases microtubule polymerization and stabilizes microtubules (Drubin and Kirschner 1986). Moreover, in primary neurons, tau regulates microtubule dynamics, organization, and growth cone ordering and growth (Biswas and Kalil 2018). However, the stabilization of neuronal microtubules by tau in mice appears redundant because tau knockout mice do not show strong microtubule impairments (Harada et al. 1994; Takei et al. 2000; Dawson et al. 2001), but double-knockout mice lacking both tau and MAP1b have greater defects in neuronal axons (Takei et al. 2000). Thus, tau binds and stabilizes microtubules and is at least partially functionally redundant with other MAPs.

The interaction of tau with microtubules suggests that it may influence axon transport. Neurons are unique, as they can have long axons and require transport of proteins and organelles over long distances. Several observations suggest that this process, termed fast axonal transport (FAT), is modulated by tau (Combs et al. 2019). For example, tau can associate in dense regions along a microtubule that hinder kinesin processivity (Siahaan et al. 2019), leading to enhanced dynein activity (Chaudhary et al. 2018). Such roles for tau may also be particularly important for the transport of components that are required for proper function and maintenance of synapses (Guedes-Dias and Holzbaur 2019).

Tau has also been proposed to have roles in protecting DNA and RNA from damage. One observation is that TUNEL staining in Tau knockout mice shows increased susceptibility to DNA and RNA damage during heat stress (Violet et al. 2014). Similarly, knockdown of tau in primary neurons increases double-stranded breaks induced by etoposide (Asada-Utsugi et al. 2022). This role may be direct, as tau has been observed in the nucleolus of cultured cells (Bou Samra et al. 2017; Maina et al. 2018), and in mice, 1N isoforms of tau are enriched in soluble nuclear fractions (Liu and Götz 2013). Such a role might be relevant because tau has nanomolar affinity for DNA (Wei et al. 2008) and RNA (McMillan et al. 2023). Thus, a less explored role for tau is in the regulation of nucleic acids.

### Disease-associated mutations of tau

There are >50 missense-coding mutations in *MAPT*, several of which are causative of severe FTD (Strang et al. 2019). These missense-coding mutations can contribute to disease through several mechanisms that generally lead to increases in tau aggregation. For example, mutation of P301S/L increases the ability for tau to aggregate as proline residues inhibit β-sheet formation (Strang et al. 2019). Furthermore, several disease-linked tau missense mutations lead to impaired microtubule binding and reduced effects on polymerization in vitro, which may increase the concentration of free tau available for aggregation (Strang et al. 2019). Interestingly, an S320F mutation that promotes spontaneous aggregation may work by both decreasing tau–microtubule interactions and increasing exposure of necessary regions for incorporation into a fiber (Rosso et al. 2002; Chen et al. 2023a), a mechanism shared by P301L/S mutations (Chen et al. 2019).

Tau mutations can also alter the ratios of tau isoforms, which can affect aggregation and disease development. The missense mutation S285R increases inclusion of exon 10, resulting in higher expression of 4R tau (Ogaki et al. 2013). Additionally another class of mutations in *MAPT* are silent or noncoding variants causing increased inclusion of exon 10, resulting in higher expression of 4R tau (D'Souza et al. 1999; Spillantini et al. 2000; Stanford et al. 2000; Skoglund et al. 2008). Many of these noncoding mutants are in intron 10 and cause destabilization of a stem–loop near the exon 10 splice site, which leads to increased inclusion and disruption in the balance of 3R and 4R tau isoforms, contributing to pathology (Qian and Liu 2014; Buchholz and Zempel 2024).

## Tau structures in disease

Primary tauopathies, such as PSP and CBD, are classified based on the primary pathological feature of tau deposition. Although secondary tauopathies also feature tau aggregation, this occurs subsequent to additional insults or pathological features such as the accumulation of amyloid-β (Aβ) plaques in AD (Chung et al. 2021). Secondary tauopathies also include chronic traumatic encephalopathy (CTE) and subacute sclerosing panencephalitis (SSPE), with repeated traumatic brain injury or viral infection preceding tau pathology, respectively (Bancher et al. 1996; Bloom 2014; Chen 2018). A convergent hypothesis is that these preceding insults trigger neuroinflammation, altering the neuronal microenvironment to enhance tau aggregation (see below). Nonetheless, the landscape of tauopathies encompasses a wide range of heterogeneous disorders.

**Figure 2.**
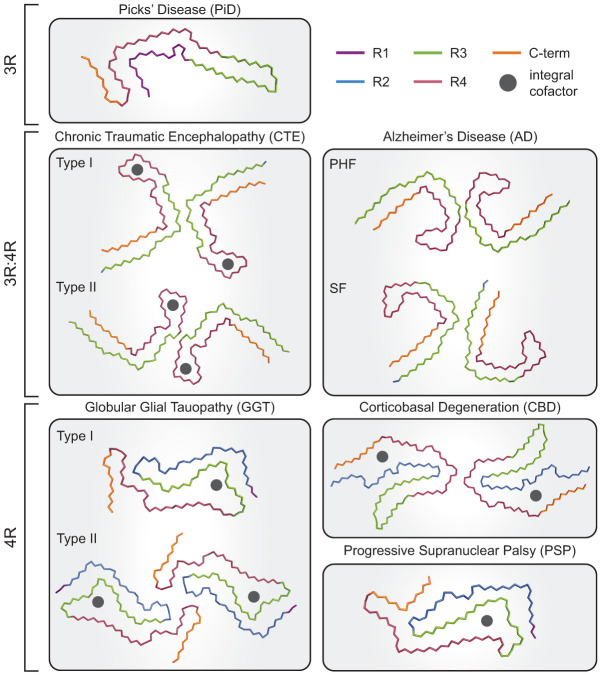
Tau fiber structures in disease. Representations of a subset of tau structures solved from several tauopathies involving inclusions of 3R, 4R, or 3R and 4R tau. Notably, solved structures are conserved across multiple patients with a given disease, and multiple structures have been solved for some tauopathies, including AD, that represent paired helical filaments (PHFs) or straight filaments (SFs) or are classified as distinct fiber types for CTE and GGT. Although tau fibers across diseases share involvement of the microtubule repeat domain region of tau, the precise amino acids involved in the core fiber differ, and their distinct structures highlight the diversity of tau fibril forms. Furthermore, several disease-relevant tau fiber structures contain nonproteinaceous cofactors that are integral to the fiber core (see Table 1).

Cryogenic electron microscopy (cryo-EM) structures from postmortem samples have shown that different tauopathies have specific tau fiber structures observed in multiple patients with a given disease (Fitzpatrick et al. 2017; Falcon et al. 2018, 2019; Zhang et al. 2020a; Shi et al. 2021) (Fig. 2; Table 1). In each case, the stable fiber core is composed of portions of the microtubule binding domain and, in some diseases, the C terminus of tau arranged in distinct geometries. Outside of the fiber core, tau remains structurally undefined and is described as a “fuzzy coat” observed in 2D class averages (Fitzpatrick et al. 2017). Interestingly, several tau fiber structures contain extra electron density that is not covalently bound to the fiber such as in CTE and CBD (Falcon et al. 2019; Zhang et al. 2020a). In CTE, the extra density is described as hydrophobic and extends through the tau fiber from end to end (Falcon et al. 2019). In CBD, the extra density is found surrounded by positively charged amino acids, suggesting a negatively charged cofactor (Zhang et al. 2020a). Despite RNA being associated with NFTs (see below), this density appears too small to be RNA. In some diseases, more than one tau fiber structure is resolved in cryo-EM. For example, in CTE, there are two related folds, and globular glial tauopathy (GGT) and PSP have three known fiber structures (Shi et al. 2021). These findings raise the possibility that distinct neuronal cell types or regions of the brain might preferentially form different tau structures. This is also suggested by spectral analyses showing that tau fibers from a single PiD patient have different properties in neurons, oligodendrocytes, and astrocytes (Yang et al. 2023).

**Table 1.** Tau structures from tauopathies

Disease	Amino acids in structure (3R/4R)	Unknown integral cofactor	Reference
Alzheimer's disease (AD)	306–378 (3R/4R)	N/A	Fitzpatrick et al. 2017
Chronic traumatic encephalopathy (CTE)	305–379 (3R/4R)	Hydrophobic	Falcon et al. 2019
Cortical basal degeneration (CBD)	274–380 (4R)	Possible polyanion	Zhang et al. 2020a
Progressive supranuclear palsy (PSP)	272–381 (4R)	Internal cofactors between N279 and G323 likely hydrophobic; K294 and D314 likely solvent; K317, K321, and K340 likely polyanion; 30 Å^3^	Shi et al. 2021
Globular glial tauopathy (GGT)	272–379 (4R)	Internal cofactors between N279 and G323 likely hydrophobic; K317, K321, and K340 likely polyanion; 50 Å^3^	Shi et al. 2021
Pick's disease (PiD)	254–378 (3R)	N/A	Falcon et al. 2018

Tau structures that have been solved by cryo-EM from various tauopathies, highlighting the amino acids involved in the fibril structures and observation of integral cofactor(s).

The diversity of tau fibers suggests that the specific structure that predominates in a given disease is dictated by the biochemical conditions and cell type where disease initiated, and that many tau fiber structures are sufficient to cause neurotoxicity. In support of this, different buffer conditions and cofactor presence can lead to several distinct tau fiber structures from the same tau isoform in vitro (Lövestam et al. 2022). Interestingly, mutations in tau do not solely dictate the resulting fiber fold, as P301S tau from two mouse models had distinct folds (Schweighauser et al. 2023). Thus, a summation of the biochemical conditions, expression, splice isoform, and intracellular conditions likely drives tau folding in disease.

## Biochemistry of tau aggregation

### Biochemical steps of tau fibrillization

In principle, the formation of tau aggregates proceeds through a series of stages, which can be overlapping. The process initiates with the formation of a “seed”—more specifically, a state of tau that shifts the equilibrium away from monomeric tau toward aggregated tau. Subsequently, the seed develops into a tau “oligomer,” which can be broadly defined as soluble tau multimers that interact with “oligomer-specific” antibodies raised against soluble, seeding-competent tau (Kopeikina et al. 2012; Lasagna-Reeves et al. 2012). Importantly, although the term “oligomer” is used to refer to an intermediate tau aggregate species, there is a need to more rigorously define the biochemical and structural properties in a consensus manner. Nonetheless, tau oligomers and resulting fibers can serve as nucleators for additional fibers. Tau fibers can also accumulate together into larger paired helical filaments (PHFs) or straight filaments (SFs), that bundle into NFTs (Crowther 1991). The formation of larger aggregates of multiple tau fibers can be understood to occur by the increased avidity of long fibers to aggregate even when individual interactions are weak. Finally, a fiber can produce secondary seeds, presumably through a process of fiber fragmentation. Each of these processes can be affected by the folding and concentration of free tau monomers.

#### Dynamics of tau folding

**Figure 3.**
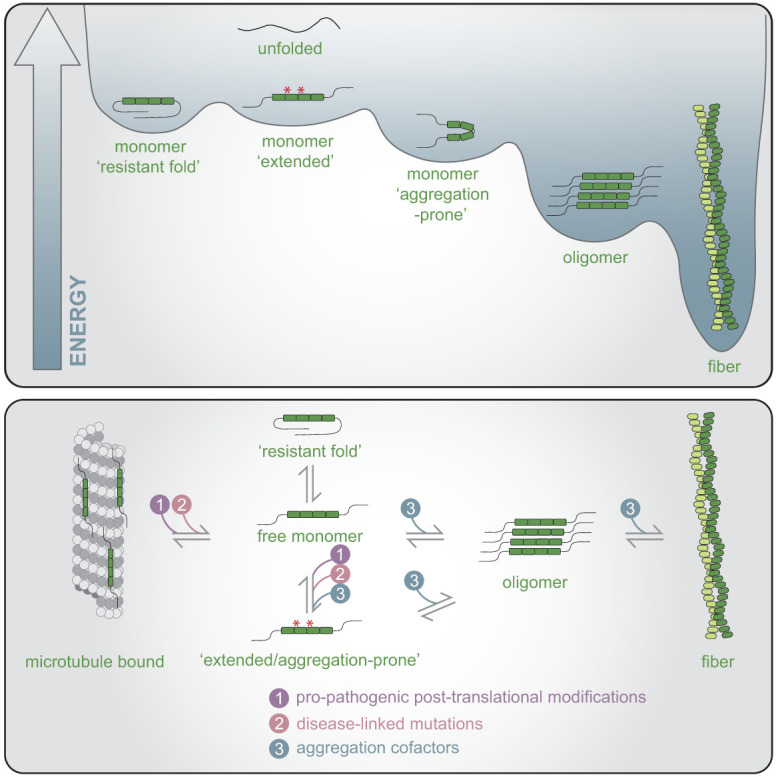
Tau folding landscape. Tau can exist in a diversity of unfolded or folded states. For example, a free tau monomer can be in a form resistant to integration into a fiber by adopting a “paperclip-like” fold or adopt an extended conformation where the PHF6 and PHF6* sequences are exposed (denoted with asterisks), which promotes incorporation into a fibrillar structure. (*Top*) Critically, pathogenic fibrillar forms of tau represent particularly energetically stable states. (*Bottom*) These states of tau exist in equilibrium, and additional influences from propathogenic post-translational modifications, disease-linked mutations, or cofactors that facilitate aggregation can affect the balance of tau states, shifting the equilibrium toward fibrillar forms.

Disordered proteins, such as tau, have no highly stable well-folded structure and instead can rapidly exchange between different monomer states with relatively low energy barriers (Trivedi and Nagarajaram 2022). This suggests that tau will be distributed between multiple states, which has key implications for tau fibrillization (Fig. 3). First, some folded states are resistant to initiating fiber formation. For example, tau can adopt a “paperclip-like” fold wherein the N and C termini fold over and block the aggregation-prone _306_VQIVYK_311_ sequence (referred to as PHF6) in the MTBR, which is the minimal sequence required to nucleate fibrilization in vitro (Li and Lee 2006; Andronesi et al. 2008; Mukrasch et al. 2009). Additionally, the different folded states of tau monomers can influence the structure of the resulting fibril. For example, the ability to form the CTE (or AD) fiber structure in vitro is dependent on different salt concentrations that stabilize specific monomer folds (Lövestam et al. 2022). Finally, as tau can exist in several folds, biochemical conditions that bias the folding of tau into the same fibrilization-prone state should increase the rate of tau aggregation.

The distribution of tau monomers into different folded states can be influenced by several factors acting in *cis* (Fig. 3). First, modification or mutation of the tau protein monomer itself can alter fibrillization propensity. This can occur in the form of pathogenic mutations such as S320F, which alters interactions within tau, reducing the “paperclip-like” fold and leading to an extended conformation with increased exposure of the PHF6 region (Chen et al. 2023a). This can also be regulated through post-translational modifications biasing folding and thereby either promoting or inhibiting fibrillization (Haj-Yahya et al. 2020; Xia et al. 2020; Boyko and Surewicz 2023).

#### Initiation of fiber formation

A key step of fibril formation is the initial formation of a seed that can then incorporate additional monomers. In the simplest model, tau proteins fold into the same conformation, bind together, and initiate a β-sheet-rich amyloid fiber, which can then grow by the addition of more tau monomers. The hexapeptide motifs _306_VQIVYK_311_ and _275_VQIINK_280_ (PHF6 and PHF6*, respectively) in the tau MTBR are both necessary and sufficient to form tau fibers in vitro (Li and Lee 2006). Thus, a fold in which these regions are exposed in multiple tau proteins would promote formation of a β-sheet-rich tau fiber in this manner (Li and Lee 2006). In fact, the exposure of these sequences can be the first observed physical change in tau that leads to tau fiber formation (Pavlova et al. 2016).

A variant model for the formation of seeds is that an individual tau monomer undergoes a chemical transition to a relatively stable conformation that is sufficient to serve as a seed for tau fibrillization. This is based on identification of a stable fraction of monomeric tau from AD brains that can serve as a seed (Mirbaha et al. 2018). A potential explanation for this observation is that this seeding-competent monomer is produced by a nonenzymatic chemical change to tau such as isomerization of proline, serine, or aspartate amino acids without breaking the backbone chain, thus altering aggregation propensity. For example, L-to-D isomerization of serine and aspartate residues in the RD of tau altered structural properties and/or fibril formation, whereas *cis*–*trans* proline isomerization was shown to promote spontaneous tau aggregation potentially via destabilizing shielding of the PHF6 motif (Chen et al. 2019; Tochio et al. 2019).

Studies using time-resolved cryo-EM demonstrated that in vitro, tau fibers can initiate with a shared filamentous intermediate that then adopts different related structures over time, with the final predominant fiber type in a population being the most thermodynamically stable under the reaction conditions (Lövestam et al. 2024). We note that these in vitro fibrillization reactions are done at high tau concentrations with no competing protein interactions. This creates conditions in which even weak interactions between tau monomers and fibers can contribute to additional fiber formation. In contrast, in cells, the tau concentration is lower and there is competition with the large excess of other components that can disrupt weak homotypic interactions (Protter et al. 2018). Thus, whether this process can occur in cells remains to be established.

#### Cofactors can mediate tau fiber formation

Cofactors are often used to increase the rate of tau fiber initiation and fibrilization in vitro and are expected to alter tau fibrilization in neurons as well (Fig. 3). Cofactors used in vitro are typically polyanions such as heparin, RNA, and some fatty acids (Dinkel et al. 2015; Fichou et al. 2018; Lövestam et al. 2022; Montgomery et al. 2023). Generally, polyanions are thought to increase tau fiber formation through neutralization of positive charges in the MTBR (Sibille et al. 2006; Fichou et al. 2019).

**Figure 4.**
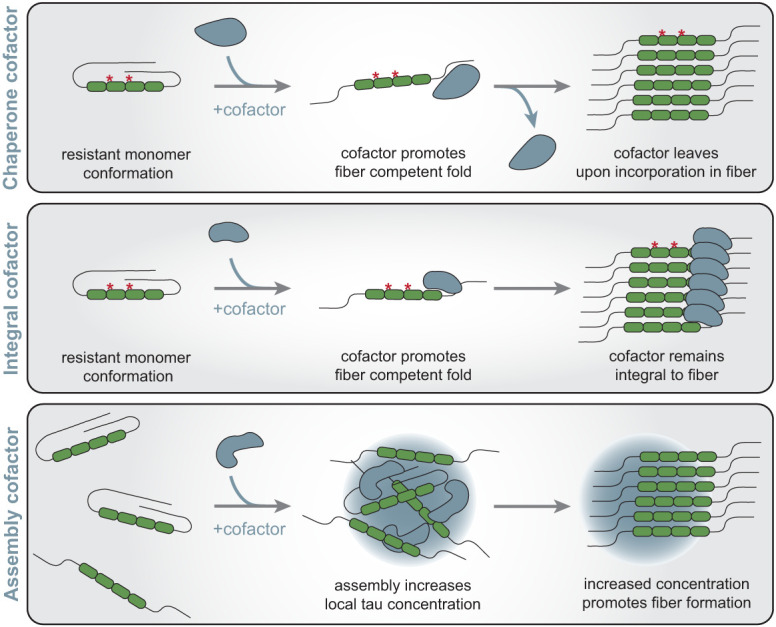
Models of tau aggregation cofactor mechanisms. Cofactors may facilitate tau aggregation through various mechanisms. First, a cofactor may serve to promote a fiber-competent fold of a tau monomer and leave once tau undergoes incorporation into a fiber. Second, a cofactor may promote a fiber-competent fold and become integral to the fiber structure. Third, a cofactor promotes tau fibrillization by increasing local concentrations of tau through the formation of assemblies through processes such as liquid–liquid phase separation.

In principle, cofactors could act in three manners to increase the rates of initiation and fibrilization of tau fibers (Fig. 4). First, cofactors could act essentially as chaperones and stabilize tau folds prone to forming fibers. For example, the ClearTau fibrilization system has shown that immobilized heparin produces tau fibers without heparin being incorporated into the fiber (Limorenko et al. 2023). Similarly, even though heparin can induce the formation of a seeding-competent tau monomer, no stable biochemical interaction is observed between tau and heparin in some experiments (Mirbaha et al. 2018). Second, cofactors may be integral to the final fiber as a structural component, as fibers prepared with heparin or RNA have been reported to depolymerize when treated with heparinase or RNase, respectively (Fichou et al. 2018). Moreover, seeding-competent tau forms from AD patient brains are reduced by treatment with RNase (Zwierzchowski-Zarate et al. 2022). Third, cofactors could act to create high local concentrations of tau monomers. For example, tau and cofactors can undergo liquid–liquid phase separation (LLPS), creating high local concentrations of tau and increasing fibrilization rates (Zhang et al. 2017; Kanaan et al. 2020). The LLPS of tau and cofactors is likely formed and maintained through several weak interactions between individual tau proteins and tau with cofactors, allowing for more protein refolding events. The chaperone and concentration effects of cofactors can be coupled such that the interaction of tau with polyanions both concentrates and stabilizes a fold that promotes fibrillization, thus enhancing the rate of initial seed formation.

It is unclear which specific cofactors in a cell can influence tau fibrilization. The structure of CBD, PSP, GGT, and CTE fibrils contain relatively small nonproteinaceous unidentified densities, indicating the presence of potential lipid or negatively charged cofactors that are integral to the fiber (Falcon et al. 2019; Zhang et al. 2020a). Other commonly used in vitro cofactors, such as RNA and heparin, may similarly influence tau fiber formation in a cellular context. In support of this, NFTs stain with RNA dyes, and tau aggregates purified from mice and cell models contain RNA with an enrichment of snRNAs and snoRNAs (Ginsberg et al. 1997; Lester et al. 2021). Additionally, endogenous heparan sulfate proteoglycans (HSPGs) have also been identified in tau tangles in AD (Su et al. 1992; Spillantini et al. 1999). There are also numerous other polyanions in cells that can stimulate tau fibrilization in vitro, but whether these can affect tau fibrilization in cells is unknown (Montgomery et al. 2023).

## Mechanisms of tau spread

The spread of tau aggregation between cells involves the generation of transmittable seeds, the transfer to other cells, and subsequent continued fiber growth, steps enabled by the “prion-like” nature of tau (Vogels et al. 2020). Several lines of evidence support that seeding-competent forms of tau can propagate across cells in a manner retaining morphological and structural characteristics defining unique “strains.” First, such tau strains, as defined by their subcellular aggregate morphology, are conserved through successive transmissions from cell to cell, cell to mouse, and mouse to cell (Sanders et al. 2014; Kaufman et al. 2016). Second, the delivery of seeding-competent tau species isolated from patient samples to the mouse brain leads to propagation to additional regions in a characteristic manner reflective of the disease origin (Boluda et al. 2015; Kaufman et al. 2016; Narasimhan et al. 2017). Third, in tau transgenic mice, fiber structures solved in early and late disease progression are identical, suggesting that propagation can maintain faithful structural replication (Schweighauser et al. 2023). It should be noted that it remains possible that, as seeds spread into new cell types or regions of the brain, differing biochemical conditions might favor a structurally different fiber fold.

**Figure 5.**
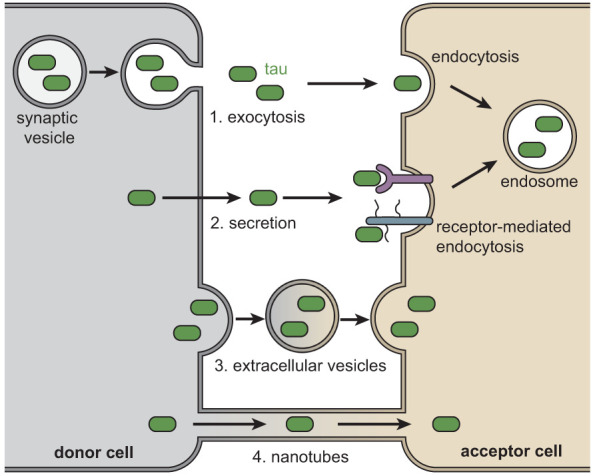
Mechanisms of intercellular tau spread. Tau species can be released and spread to additional cells through several mechanisms, including exocytosis and direct secretion, in extracellular vesicles or through tunneling nanotubes. Extracellular tau can be taken up by surrounding cells by endocytosis pathways.

Tau can be transmitted from cell to cell in several manners (Fig. 5). First, studies indicate that tau pathology can spread through synaptic connections. This was first suggested by the reliable patterning of tau pathology during Braak staging in AD, with tau affecting the entorhinal cortex at early stages and extending to synaptically connected regions such as the hippocampus and cerebral cortex at later stages (Braak et al. 2006). Other primary tauopathies also involve the sequential progression of pathology across brain regions but with different patterns than AD (Saito et al. 2004; Williams et al. 2007; Irwin et al. 2016). For example, in PSP, the pallido–nigro–luysian axis is affected by tau pathology at early stages, which then progresses to affect neocortical regions and the cerebellum (Kovacs et al. 2020). The spread of tau pathology through neuronal circuits is experimentally supported by studies in mouse models, as regions synaptically connected to the initial site of tau pathology are progressively affected (de Calignon et al. 2012; Ahmed et al. 2014). Supporting that tau can spread from neuron to neuron *trans*-synaptically in humans, tau seeding activity was observed in brain regions composed of axons and synaptic fractions (DeVos et al. 2018), and oligomeric tau labeled with conformation-specific antibodies has been identified in presynaptic and postsynaptic terminals in AD brains even in regions that do not have extensive tangle pathology (Colom-Cadena et al. 2023).

The spread of tau pathology can also occur through release of tau into the extracellular space. Tau can be detected at increased levels in the cerebrospinal fluid of AD patients (Vandermeeren et al. 1993). Furthermore, antibodies that bind extracellular tau can prevent further uptake and have been shown to reduce pathological spread in mice (Yanamandra et al. 2013; Congdon and Sigurdsson 2018). Mechanistically, tau can be secreted directly via translocation across the plasma membrane (Katsinelos et al. 2018) and subsequently taken up by surrounding cells through binding to extracellular receptors such as low-density lipoprotein receptor-related protein 1 (LRP1) and HSPG (Rauch et al. 2018, 2020). Inhibition of the LRP1 or HSPG uptake mechanisms reduces the development of tau pathology in mice, demonstrating this as a relevant mode of tau seed transmission.

Tau can also spread through vesicle-mediated release and enter cells through endocytosis or membrane fusion (Saman et al. 2012; Mulcahy et al. 2014; Wang et al. 2017b; Katsinelos et al. 2018). In support of this, the loss of a neuronal isoform of bridging integrator 1 (BIN1), an AD risk gene and negative regulator of clathrin-mediated endocytosis, increases the propagation of tau aggregation (Calafate et al. 2016). Furthermore, the delivery of exosome-contained tau facilitates the propagation of pathology in model systems (Polanco et al. 2016; Winston et al. 2019). Recent results also suggest that the release of tau seeds transmitted in extracellular vesicles can be regulated by Arc (Tyagi et al. 2024).

Last, tau has also been shown to transit through tunneling nanotubes between cultured cells; however, the contributions of this transport mode for tau in in vivo models have not yet been reported (Tardivel et al. 2016).

## Intracellular influences on tau aggregation

**Figure 6.**
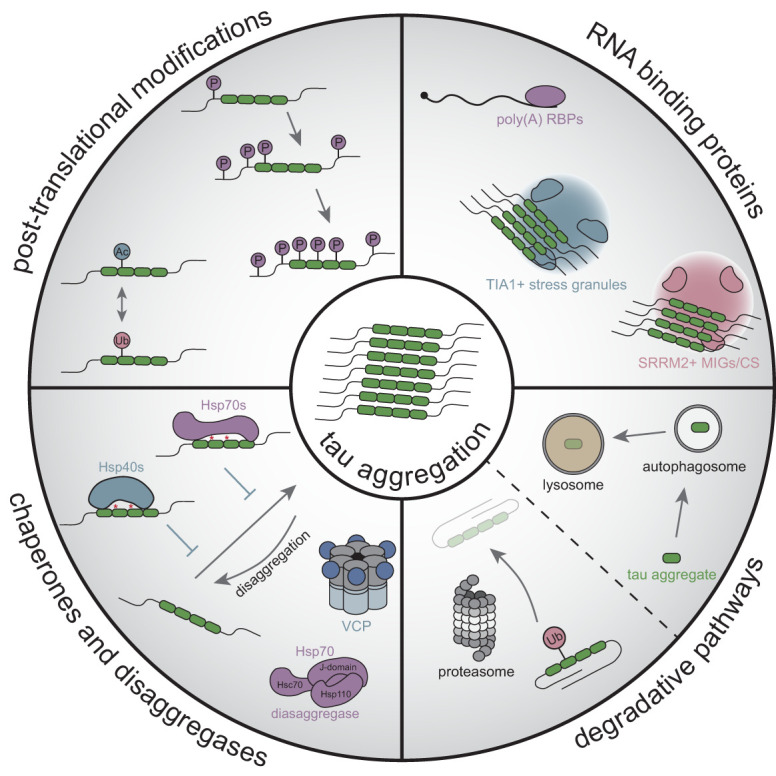
Cellular influences on tau aggregation. Several cellular pathways can influence the formation of tau aggregates. These include post-translational modifications of tau such as phosphorylation (P), acetylation (Ac), and ubiquitination (Ub), which can modulate stability, microtubule binding, or propensity to aggregate. Also, several RNA binding proteins (RBPs) have been linked to tau pathology, such as poly(A) RBPs, or those enriched in cellular assemblies, such as stress granules, mitotic interchromatin granules (MIGs), or cytoplasmic speckles (CSs), which have been proposed to promote tau aggregation. Additionally, chaperones such as Hsp40s and Hsp70s serve to mitigate tau misfolding, whereas the Hsp70 and VCP disaggregase machinery can disassemble tau aggregates. Last, cellular degradative pathways such as the ubiquitin/proteasome and autophagy/lysosomal machinery are key regulators of tau clearance and thus can also influence aggregation.

Tau aggregation can be altered by several intracellular factors and processes that impact folding, fibrillization, and spread (Fig. 6). First, post-translational modification of tau can have key regulatory effects such as altering the folding landscape and aggregation propensities. Second, the cumulative effects of numerous intracellular tau-interacting partners, including microtubules, can change the available and folded states of soluble tau. Last, the extent of tau aggregation is altered by active cellular processes protecting against the formation of toxic aggregates such as chaperone and disaggregase pathways.

### Post-translational modification and processing

Within a cell, tau is subject to extensive post-translational modifications (PTMs). Tau PTMs are of significant interest, as certain modifications are enriched in tau aggregates and accumulate with the progression of pathology in tauopathies such as AD (Wesseling et al. 2020). For modifications associated with pathogenic tau, it is important to consider whether they accrue during disease and thus are a feature but do not directly affect the pathogenic cascade or whether they alter the biochemical properties of tau in a manner that actively modulates tau aggregation. In this section, we discuss a few notable examples of tau PTMs, a topic that has previously been extensively reviewed (Alquezar et al. 2021).

#### Phosphorylation

The phosphorylation of tau is the most extensively studied post-translational modification spurred on by the finding that fibrillar forms of tau present in AD are “hyperphosphorylated” (Grundke-Iqbal et al. 1986). Furthermore, phosphorylation of tau at certain epitopes is often used as a marker of pathology (Götz et al. 2010). Several kinases and phosphatases that act on tau have been identified and investigated for contributions to pathology and therapeutic potential, as reviewed previously in more detail (Martin et al. 2013a,b). A key example that is supportive of phosphorylation playing a role in pathology includes the knockdown or activation of cyclin-dependent kinase 5 (CDK5), a major tau kinase, which reduced or increased aggregation of tau in mouse models, respectively (Noble et al. 2003; Piedrahita et al. 2010; Castro-Alvarez et al. 2014). Similarly, inhibition of a related tau kinase, glycogen synthase kinase 3 (GSK-3), reduces tangle formation in transgenic mice (Hurtado et al. 2012). However, such kinases often phosphorylate tau at multiple sites and have many other cellular targets, warranting more direct biochemical investigation of the effects of tau phosphorylation specifically.

Despite the observation that tau aggregates are hyperphosphorylated, the effect of tau phosphorylation on fibrilization and aggregation is more nuanced. For example, while phosphorylation in the MTBR leads to a reduction in microtubule affinity, it also inhibits aggregation kinetics in in vitro assays and reduces seeding in cells (Kellogg et al. 2018; Haj-Yahya et al. 2020; Powell et al. 2024). Although such a phosphorylation modification may not directly increase fibrillization rates, phosphorylation could modulate the concentration of cellular tau unbound to microtubules available for aggregation. In contrast, phosphorylation of residues in the PRR have been directly linked to increased aggregation in vitro and in cells (Despres et al. 2017; Xia et al. 2020). Ultimately, the effects of tau phosphorylation are site-specific, and the mechanisms through which it can influence aggregation may be dependent on the modified region of tau.

Interestingly, increases in specific phosphorylated residues of tau have been developed as biomarkers measurable in CSF or plasma from AD patients but are not observed in primary tauopathies, emphasizing that they may more likely reflect Aβ pathology (Zetterberg 2022; Therriault et al. 2023). Furthermore, while increased phosphorylation of tau is observed in insoluble forms in AD, these modifications can also occur in healthy brains (Wegmann et al. 2021). Thus, while phosphorylation of tau is a feature of pathology, this does not indicate that phosphorylated tau is strictly toxic, and attention must be paid to the precise modifications and the mechanistic manners in which they may influence aggregation.

#### Acetylation

Abnormal acetylation of tau at specific residues has been reported in AD and related tauopathies associated with aggregation (Min et al. 2010; Cohen et al. 2011; Irwin et al. 2012; Tracy et al. 2016; Wesseling et al. 2020). In vitro assays indicate that tau acetylation can promote fibrillization (Cohen et al. 2011). Additionally, animal models with acetyl-mimetic mutants of tau show that acetylation can inhibit protein turnover, increasing tau levels (Min et al. 2010), and play a role in impaired synaptic plasticity (Tracy et al. 2016). The deacetylase silent information regulation 2 homolog 1 (SIRT1) is linked to tau pathology progression in animal models, with conditions of reduced acetylation having less tau aggregation (Min et al. 2010, 2018). Alternatively, acetylation at KXGS motifs in the MTBR of tau is inversely related to phosphorylation and reduces the propensity for aggregation, highlighting both the interplay of multiple PTMs and the differing effects of acetylation of specific residues (Cook et al. 2014; Carlomagno et al. 2017). Acetylation also competes with ubiquitination modification of lysine residues and thus can be a key determinant of tau stability.

#### Ubiquitination

Ubiquitination is a critical cellular mechanism for regulating protein turnover through degradative pathways such as the ubiquitin–proteasome machinery or autophagy as discussed below. In the AD brain, tau polyubiquitination is increased, consistent with impairments in protein homeostasis (Abreha et al. 2018). Immunostaining of AD brains shows that NFTs of tau are ubiquitin-positive, but this modification follows N-terminal processing, suggesting that it is a consequence of tau aggregation rather than causal (Mori et al. 1987; Iwatsubo et al. 1992; Morishima-Kawashima et al. 1993). Ubiquitination can also alter tau biochemical properties, as this modification can inhibit tau fibrilization and phase separation in vitro. (Trivellato et al. 2023).

#### Other post*-*translational modifications

Tau is also subject to many additional post-translational modifications such as methylation, SUMOylation, glycosylation, and others, though the effects on disease processes are not as widely studied (Alquezar et al. 2021). Collectively, with regards to PTMs and effects on tau aggregation and pathology, they must be validated in a residue-specific manner in model systems. Furthermore, the complex integration of the vast number of potential PTMs of tau occurring in a cell will inform whether a given tau protein is favored to initiate or incorporate into a tau aggregate.

### RNA and RNA binding proteins

Tau expression is regulated at the post-transcriptional level by RNAs and RNA binding proteins (RBPs). For example, exon 10 inclusion in *MAPT* is regulated by the serine- and arginine-rich splicing factor 2 (SRSF2), and loss of function of SRSF2 results in increased expression of 4R tau (Qian et al. 2011; Chen et al. 2014). miRNAs also regulate tau expression and post-translational modifications, though whether these are through direct effects on tau or through alteration of cellular pathways that regulate tau is unknown (Boscher et al. 2020).

Tau protein has also been observed to interact with RNAs and RBPs. RNA can be a cofactor for tau fibrillization in vitro, and tau aggregates in cell and mouse models contain RNA, most notably snRNAs and snoRNAs (Kampers et al. 1996; Lester et al. 2021). Characterization of tau and RNA interactions in cells has also shown particular association with tRNAs (Zhang et al. 2017). Moreover, multiple studies have reported the enrichment of RBPs in NFTs from tauopathy patient tissue and localization of RBPs in tau aggregates in model systems (Kavanagh et al. 2022). For example, U1 small nuclear ribonucleoprotein (snRNP) components were identified in tau inclusions in AD (Bai et al. 2013).

In some cases, RBPs have been proposed to affect the development of tau pathology and/or toxicity. Suppressors of tau toxicity in *Caenorhabditis elegans* models identified several poly(A) RBPs and deadenylases—including suppressor of tauopathy 2 (sut-2), polyadenylate-binding protein 2 (pabp-2), and poly(A)-specific ribonuclease (parn-2)—that modulate pathological tau (Guthrie et al. 2009; Wheeler et al. 2019; Kow et al. 2021). Deletion of the mammalian homolog of sut-2 (MSUT2) in the mouse brain rescues tau aggregation and neuronal loss, whereas overexpression exacerbates pathology (Wheeler et al. 2019). PABP-2/PABPN1 and MSUT2 are codepleted in AD brain sections, which stratifies with earlier onset, supporting a role for these RBPs in human disease (Wheeler et al. 2019). Relatedly, the loss of poly(A) deadenylase parn-2/TOE1 leads to increased tau pathology in *C. elegans* and correlates with MSUT2 expression and increased tau pathology in AD patient brains (Kow et al. 2021). These results suggest that the regulation of RNAs by poly(A)-related factors is important for tau pathology, though further investigation is necessary to establish the precise molecular mechanisms.

The spliceosome component serine/arginine repetitive matrix 2 (SRRM2) colocalizes with tau inclusions in tauopathy disease tissues (Lester et al. 2021; McMillan et al. 2021). Interestingly, SRRM2 and associated nuclear speckle protein pinin (PNN) not only mislocalize to cytoplasmic tau inclusions but also define cytoplasmic assemblies rich in polyserine motifs that are sites of tau aggregation in cellular models (Lester et al. 2023). In support of a role in pathobiology, increased levels of polyserine exacerbate tau pathology in both cell lines and mouse models (Lester et al. 2023; Van Alstyne et al. 2024).

The stress granule RBP T-cell intracellular antigen 1 (TIA1) has also been shown to colocalize with pathogenic tau in disease (Vanderweyde et al. 2012). Stress granules are cytoplasmic assemblies of untranslating messenger ribonucleoproteins (mRNPs) that form when translation initiation is reduced (Protter and Parker 2016). TIA1-containing stress granules are proposed to facilitate tau aggregation, and the reduction of TIA1 levels reduces tau pathology in a mouse model (Apicco et al. 2018). Two possible mechanisms for this effect have been proposed. First, TIA1 is proposed to directly interact with tau and increase the formation of oligomeric and toxic tau (Apicco et al. 2018; Jiang et al. 2019; Ash et al. 2021). Additionally, TIA1 in microglia is proposed to regulate the inflammatory response in a manner that reduces tau toxicity (Webber et al. 2024).

Stress granule RBPs have also been linked to tau aggregation through additional mechanisms. Ras GTPase-activating protein-binding protein 2 (G3BP2), an integral component of stress granules, was shown to chaperone tau via an RNA-independent mechanism by binding to soluble forms and preventing oligomerization (Wang et al. 2023). Conversely, heterogenous nuclear ribonucleoprotein A2/B1 (hnRNPA2B1) has been proposed to promote tau pathology through mediating association with methyl-6-adenosine (m^6^A) RNA species (Jiang et al. 2021). However, there are conflicting reports on whether hnRNPA2B1 colocalizes with tau aggregates in postmortem tissues (Jiang et al. 2021; Kavanagh et al. 2024). Nevertheless, a possible role for m^6^A RNA modifications is supported by genes promoting m^6^A modification being required for increased T22 antibody-positive oligomeric tau in a CRISPRi-based screen of iPSC-derived neurons (Samelson et al. 2024).

### Chaperones and disaggregases

The chaperone network is the key cellular defense to surveille and prevent protein misfolding that can lead to aggregation and proteotoxicity. Several classes of molecular chaperones are linked to tau, including the core machinery that uses ATP hydrolysis to control reversible binding to client proteins as well as cochaperones, which enhance activity and help determine substrate specificity. In addition, cellular machinery such as disaggregases can extract tau from aggregates.

#### Molecular chaperones

Core chaperone machinery, including heat shock protein 70 (Hsp70) proteins, has been linked to tau. Staining of hippocampal sections from AD brains showed that phosphorylated tau and Hsp70 were inversely related, suggesting that it may play a protective role in disease (Dou et al. 2003). In support of this, in vitro and cellular assays show that Hsp70 can associate with tau, promote degradation, and prevent aggregation (Dou et al. 2003; Petrucelli et al. 2004; Shimura et al. 2004; Patterson et al. 2011; Voss et al. 2012). Although Hsp70 can act at earlier stages in chaperoning de novo protein folding, Hsp90 may act on later intermediates and has more restricted client selectivity (Karagöz et al. 2014). Hsp90 can bind to the Hsp70–tau complex via a broad region encompassing the MTBR and can elicit opposing effects of either stabilizing or promoting degradation depending on the cofactors involved (Dickey et al. 2007; Karagöz et al. 2014). In support of a pathogenic role where Hsp90 stabilizes disease-relevant tau, Hsp90 inhibition can selectively increase degradation of pathogenic tau in cellular and mouse models (Dickey et al. 2007; Luo et al. 2007).

The Hsp40 J domain-containing (DnaJ) proteins, which function as cochaperones with Hsp70s by promoting substrate binding and activating ATP hydrolysis, have also been linked to tau. Multiple DnaJ proteins from classes A, B, and C, which differ in their conserved domains, have been shown to bind various tau species and modulate aggregation (Ryder et al. 2022). For example, DnaJB1 has been shown to suppress tau aggregation and bind to tau fibers but not monomers in vitro (Irwin et al. 2021). Conversely, DnaJC7 preferentially binds a native fold of tau, suppressing tau aggregation by blocking PHF6* accessibility and potentially preventing incorporation into a fiber (Hou et al. 2021). DnaJA2 also prevents tau aggregation and has affinity for a broader range of tau forms, including monomeric and fibrillar forms of tau in vitro (Mok et al. 2018; Irwin et al. 2021). In line with distinct mechanisms of action and affinity of different DnaJ proteins, activity toward tau aggregation is not shared across all J domain-containing proteins, underlining a degree of specificity (Perez et al. 2023).

Additional chaperones affecting tau include clusterin, a secreted protein identified as a risk gene for AD through genome-wide association studies (GWASs) (Harold et al. 2009; Lambert et al. 2009; Wojtas et al. 2020). Interestingly, clusterin appears to directly affect tau pathology, as it colocalizes with pathological tau accumulations in tauopathies, and knockout of clusterin in tau transgenic mice exacerbates pathology (Wojtas et al. 2020). Also, Hsp27 can interact with tau, and increased expression in tau transgenic mice can reduce tau levels (Abisambra et al. 2010). The cochaperone BCL-associated anthanogene2 (BAG2) has also been shown to promote ubiquitin-independent proteasomal degradation of tau in complex with Hsp70 (Carrettiero et al. 2009). Additionally, the E4 isoform of apolipoprotein (ApoE), a pathological chaperone that regulates amyloid-β aggregation (Wilhelmus et al. 2007), is a strong risk factor for developing AD and also exacerbates tau pathology in mouse models (Shi et al. 2017). The folding of tau and its propensity to fibrillize are likely affected by additional protein chaperones that are yet to be identified.

#### Cellular disaggregase machinery

Cellular disaggregases serve to disassemble misfolded or aggregated protein complexes and thus can play an important role in tau aggregation. As such, the Hsp70 disaggregase machinery—consisting of Hsc70-specific J domain-containing proteins and heat shock protein 110—has been shown to process recombinant and AD-extracted tau fibrils in vitro (Nachman et al. 2020). Additionally, valosin-containing protein (VCP) is an AAA^+^ ATPase that can extract ubiquitinated proteins from larger protein complexes or membranes through ATP-dependent unfolding (Meyer and van den Boom 2023). VCP can disassemble tau aggregates in cellular models, and a hypomorphic mutation is linked to the formation of tau aggregates in vacuolar tauopathy (Darwich et al. 2020; Saha et al. 2023). Furthermore, VCP levels are reduced in postmortem AD patient brains, where they inversely correlate with a marker of phosphorylated tau, and VCP overexpression in a viral-driven tau mouse model suppresses tau pathology (Giong et al. 2024). However, studies investigating disaggregase mechanisms in cell models have shown that this activity can produce smaller but still seeding-competent species of tau and thus might increase spread (Nachman et al. 2020; Saha et al. 2023). Collectively, these findings highlight disaggregase machinery as an important regulator of the equilibrium of tau states.

### Degradative pathways for tau

Cellular mechanisms regulating tau protein turnover also play a key role in pathology. Tau isolated from AD brains has been shown to be ubiquitinated at several lysine residues within the MTBR that are primarily Lys-48 polyubiquitinated—the main signal for proteasomal degradation (Morishima-Kawashima et al. 1993; Cripps et al. 2006). Tau also associates with ubiquitin–proteosome system (UPS) components. In particular, the C-terminal heat shock protein 70-interacting protein (CHIP) is an E3 ligase that can ubiquitinate tau, and the deletion of CHIP in mice overexpressing mutant human tau leads to an accumulation of phospho-tau species (Petrucelli et al. 2004; Shimura et al. 2004; Dickey et al. 2006). Additional ubiquitin ligases that may modify tau include Praja ring finger ubiquitin ligase 1 (Praja-1) (Aoki et al. 2024) and cullin-5 (CUL5) (Samelson et al. 2024). Furthermore, tau deubiquitinases such as OUT domain ubiquitin aldehyde binding 1 (Otub1) and ubiquitin-specific peptidase 10 (Usp10) can promote tau accumulation and aggregation (Wang et al. 2017a; Wei et al. 2022).

The autophagy–lysosomal pathway (ALP) is another cellular degradative pathway linked to tau. Interestingly, disease-associated mutations, phosphomimetics, and acetylation reduce the ability of tau to be degraded through chaperone-mediated autophagy (Caballero et al. 2018, 2021). Chemical stimulation of autophagy with a mammalian target of rapamycin (mTOR) activator in transgenic mice expressing P301S tau resulted in reduced insoluble tau in the brain and rescued neuronal loss in the cortex (Schaeffer et al. 2012). Additionally, the knockout of a key autophagy factor, Atg7, in postnatal neurons led to the accumulation of phosphorylated tau (Inoue et al. 2012). Last, an accumulation of autophagic vesicles was observed in brains from AD patients, supporting the involvement of ALP in neurodegeneration (Nixon et al. 2005).

Thus, the pathways mediating turnover of tau can modulate aggregation and pathology, underlining that strategies to reduce tau levels can be therapeutically effective.

## Contributions of glial cells to tau pathology

Within the context of the brain, neurons exist in homeostasis with glia that have critical support and maintenance roles. Such effects on the physiological state of neurons can modulate tau pathology (Perea et al. 2020; Odfalk et al. 2022). GWASs in AD and, to a lesser extent, PSP have identified several risk genes expressed exclusively or primarily in glial cells such as microglia and astrocytes, emphasizing the importance of nonneuronal cell types (Karch and Goate 2015; Gallo et al. 2023; Farrell et al. 2024). In addition, genetic manipulation of nonneuronal cell types can alter the course of tau disease in mouse models, highlighting the importance of intercellular interactions (see below). An overarching principle is that the neuronal microenvironment (defined as the interactions with neighboring cells and extracellular signals such as cytokines) alters the physiology of neurons in a manner that modulates disease.

### Contributions of microglia

Microglia are the predominant immune cells in the central nervous system, involved in maintaining homeostasis through phagocytosis of damaging particles and mediating neuroinflammatory responses through the secretion of factors such as cytokines (Colonna and Butovsky 2017). Microglial activation is associated with tau pathology in disease contexts and animal models (Yoshiyama et al. 2007; Pascoal et al. 2021). The role of microglia in promoting tau pathology is supported by the observation that in a viral-based mouse model of rapid tau propagation and in tau transgenic mice, the depletion of microglia through inhibition of colony-stimulating factor 1 receptor (CSF1R) reduces inflammatory gene signatures and tau pathology (Asai et al. 2015; Mancuso et al. 2019).

There are multiple mechanisms through which microglia may modulate tau pathology at various stages in disease. Importantly, microglia may first take on a protective role through the uptake of tau seeds, potentially reducing spread; however, if they are unable to accommodate pathogenic tau, they can become maladaptive (Luo et al. 2015; Hopp et al. 2018). To enable their normal role, microglia are highly adaptive to environmental signals to mount appropriate responses and have been shown to adopt disease-associated transcriptional programs linked to tau pathology (Deczkowska et al. 2018). This disease-associated microglia (DAM) transcriptional profile leads to the upregulation of inflammatory genes that can be detrimental to neuronal homeostasis (Odfalk et al. 2022).

Specific cellular pathways have been implicated in the microglia-mediated contributions to tau pathology. Evidence for activation of the classical complement pathway involved in innate immunity is observed in AD and tauopathy patients (Litvinchuk et al. 2018). In particular, increases in expression of complement component 3 (C3) and the C3a receptor (C3aR) correlate with disease severity, and deletion of C3aR in tauopathy mice attenuated pathology and normalized the DAM transcriptional signature (Litvinchuk et al. 2018). Thus, activation of the classical complement cascade can serve as a critical mode of cross-talk between neurons, astrocytes, and microglia, modulating the neuroinflammatory state and exacerbating tau pathology.

Other components of the innate immune system can play a role in the development of tau pathology. The nucleotide-binding domain leucine-rich repeat (NLR)-containing family pyrin domain containing 3 (NLRP3) inflammasome is elevated in tau-linked FTD patients, and genetic loss of constituents NLRP3 or apoptosis-associated speck-like protein containing a caspase recruitment domain (ASC) ameliorates tau pathology occurring downstream from Aβ in model mice (Ising et al. 2019). Tau monomers, oligomers, or fibrils can activate the NLRP3 inflammasome in primary microglia, which correlates with increased expression of proinflammatory cytokines (Ising et al. 2019). Pathogenic tau can also activate the cyclic GMP–AMP synthase–stimulator of interferon genes (cGAS–STING) protein pathway in microglia, leading to an interferon response (Jin et al. 2021; Udeochu et al. 2023). Last, exposure to tau fibrils can activate the nuclear factor κ light chain–enhancer of activated B cells (NF-κB) pathway in microglia, and inactivation can reduce tau spread and cognitive deficits (Wang et al. 2022). Thus, microglial-mediated neuroinflammation is a key feature of tau pathology that can exacerbate neuronal dysfunction.

### Contributions of other glia and infiltrating cells

Astrocytes can also influence the progression of tau pathology. Astrocytes can take up tau (Perea et al. 2019) and participate in the spread of tau between neurons (Eltom et al. 2024). Similar to microglia, astrocytes can change their gene expression profiles in response to insults and, in such an activated state, can contribute to disease progression (Escartin et al. 2021). Evidence that astrocytes can specifically affect tau disease progression comes from astrocyte-specific ablation of ApoE4 (a GWAS-linked AD gene) in a P301S tau transgenic mouse model, which reduced neurodegeneration (Wang et al. 2021a). In addition, neurotoxic effects of astrocytes are marked by C3 positivity (Liddelow et al. 2017). Interestingly, elevations in classical complement genes in tau transgenic mice were most pronounced in astrocytes, and deletion of C3 in tau transgenic mice alleviated aspects of tau pathology (Wu et al. 2019).

Recent results also suggest that T cells can affect tau-mediated neurodegeneration. Interestingly, T cells can enter the brain during normal aging (Gemechu and Bentivoglio 2012). Furthermore, regions of tau pathology and T cells in the brain show correlation in tau transgenic mice and AD patient brains (Chen et al. 2023b). Genetic ablation or inhibition of T cells in a tauopathy mouse model prevented tau-mediated neurodegeneration (Chen et al. 2023b). However, the precise mechanisms in which T cells act on tau pathology remain to be determined.

## Cellular toxicity of tau

### Gain-of-function toxicity of tau

In principle, tau could lead to toxicity due to the loss of normal functions and/or the gain of toxicity related to tau's propensity to misfold, aggregate, and spread pathology. Although the basis for tau toxicity is not fully understood, several observations suggest that gain of toxic mechanisms are important. First, although there is some evidence for microtubule deficits induced by tau knockout in mouse models, they are relatively minor, likely due to compensation by other microtubule-associated proteins (Harada et al. 1994; Takei et al. 2000; Dawson et al. 2001). Similarly, tau knockout animals show mild behavioral and neurological phenotypes (Ikegami et al. 2000; Dawson et al. 2001; Roberson et al. 2007; Lei et al. 2012). Thus, loss of function of tau is not the main source of toxicity. Moreover, transgenic mice or rats that overexpress human tau with disease-associated mutations reproduce key aspects of disease, such as the formation of insoluble tau species, synaptic dysfunction, neuronal loss, and cognitive defects (Ramsden et al. 2005; Yoshiyama et al. 2007). Additionally, individuals with extra copies of the *MAPT* gene can develop early-onset dementia clinically similar to AD, consistent with a gain-of-toxicity mechanism (Wallon et al. 2020). Furthermore, generation of a transgenic model expressing a proaggregation mutant form of tau led to neurotoxicity, which was abolished due to proline point mutations that prevent β structure despite similar expression levels (Mocanu et al. 2008). Thus, in the simplest model, gain of toxicity is related to the propensity to form misfolded tau species.

### Toxicity of oligomeric or fibrillar forms of tau

An unresolved issue is the specific form(s) of tau that mediate toxicity. In principle, toxicity could be driven by particular monomeric, oligomeric, and/or larger fibrillar forms of tau. Because tau oligomers can lead to subsequent tau fibrilization, a parsimonious model is that oligomeric tau is a smaller version of the same cross-β structure seen in the larger fiber. However, why such oligomers would exhibit specific immunoreactivity to developed oligomeric-specific antibodies is unclear, and the precise biochemical nature of oligomeric tau is often poorly defined.

Nonetheless, several lines of evidence support a toxic role for these soluble, oligomeric tau species. Regional analysis shows that synaptic and neuronal loss can occur prior to the detection of NFTs in tau transgenic mouse models (Spires et al. 2006; Yoshiyama et al. 2007), and *Drosophila* models exhibit tau-induced neurotoxicity in the absence of fibrillar tau aggregates (Cowan et al. 2011; Sun and Chen 2015). Furthermore, the stereotaxic injection of tau oligomers (characterized to be primarily dimers or trimers based on molecular weight), but not fibrils or monomers, causes synaptic defects and cognitive impairments in wild-type mice (Lasagna-Reeves et al. 2011). Why might soluble, misfolded tau species be more toxic? One consideration is that relative to larger fibers, oligomeric tau species may have an increased ability to enter and spread between cells. This is particularly relevant when delivered extracellularly, as lower-molecular-weight oligomeric species may exhibit more toxicity due to increased uptake compared with larger, insoluble tau fibers. It is also possible that tau contained in larger fibrillar structures may be less accessible than smaller oligomeric forms for gain-of-function toxic associations with other factors within the cell.

The toxicity of soluble oligomeric tau species has led to a proposed model in which fibrillar tau aggregates are a sink for the main toxic species and perhaps play a protective role in disease (Kopeikina et al. 2012). In support of this, the accumulation of tau reactive with an antibody (PHF1) raised against paired helical filaments from patient brains does not necessitate neuronal dysfunction and loss in regions such as the visual cortex and striatum, though these neuronal populations may have differential vulnerabilities to insoluble tau accumulations (Spires et al. 2006; Kuchibhotla et al. 2014). Additionally, mouse models using conditional expression of human mutant tau have shown that turning off transgene expression after onset of pathology can prevent neuronal loss and improve cognitive function despite increases in the proportion of PHF1-positive neurons (SantaCruz et al. 2005; Spires et al. 2006). Although the presence of large tau accumulations may be characteristic of a late stage in the tau pathogenic cascade, it does not mean that higher-order fibrillar aggregates are innocuous. Large tau aggregates may have distinct mechanisms of toxicity such as the sequestration of RBPs (Bai et al. 2013; Drummond et al. 2020; Lester et al. 2021). Additionally, with the active disassembly mechanisms discussed earlier, larger tau aggregates may be a source of more seeding-competent tau, and evidence suggests that tau aggregates in vivo are somewhat dynamic, as they can be reversed if tau expression is reduced (DeVos et al. 2017; Saha et al. 2023).

Taken together, these findings highlight that understanding the precise toxic forms of tau is a poorly understood but critical and complex issue. One anticipates that multiple different forms of tau can exert toxicity through distinct mechanisms, and the equilibrium of different forms as well as the broader cellular context will likely impact the nature of the toxic response.

### Cellular pathways implicated in tau-mediated neuronal dysfunction

Pathogenic tau has been suggested to elicit neuronal dysfunction and degeneration through the dysregulation of multiple cellular pathways. It is likely that tau-driven neuronal degeneration is a consequence of multiple modes of dysregulation acting either in an additive manner or at different stages of disease progression.

#### Synaptic dysfunction and loss

The loss of synapses is a feature of primary and secondary tauopathies and correlates with the regional specificity characteristic of disease (Bigio et al. 2001; Lipton et al. 2001; Clare et al. 2010). Furthermore, impairments in synaptic transmission occur prior to significant tangle formation in tauopathy model mice (Yoshiyama et al. 2007). Although tau is primarily located in axons under normal conditions, disease-associated missorting of tau to the somatodendritic space can facilitate toxicity by disrupting normal roles and/or promoting other spatially restricted interactions (Gendron and Petrucelli 2009). Pathological tau localization to synaptic compartments occurs in AD patient tissue and tauopathy mouse models, where it is linked to both presynaptic and postsynaptic dysfunction (Henkins et al. 2012; Sahara et al. 2014).

In cultured neuronal models, tau interacts with synaptic vesicle proteins, and tau with FTD-associated mutations can slow synaptic vesicle mobility (Zhou et al. 2017; Tracy et al. 2022). Synaptogyrin-3 mediates association of tau with presynaptic vesicles, and its reduction can rescue tau-dependent defects in neurotransmitter release in primary cultured neurons (McInnes et al. 2018). In addition to directly altering synaptic vesicles, tau can regulate the axonal transport of cargos important for synaptic function through associations with kinesin and dynein motors. In models overexpressing various forms of tau, impairments in the axonal transport of mitochondria and other organelles have been observed (Stamer et al. 2002; Shahpasand et al. 2012). Also, disruption in the transport of peroxisomes due to tau overexpression sensitizes cells to oxidative insults (Stamer et al. 2002; Kopeikina et al. 2012). Thus, the lack of key molecules or organelles at distal synapses could also exacerbate synaptic defects (Gendron and Petrucelli 2009).

At the postsynapse, targeting of the kinase Fyn, which regulates N-methyl-D-aspartate (NMDA) receptor activation, occurs in a tau-dependent manner, and disruption through the knockout or relocalization of tau reduces Aβ-mediated excitotoxicity (Ittner et al. 2010). Thus, increased tau in postsynaptic compartments may also lead to increased vulnerability to NMDA-mediated excitotoxicity, and in support of this, the expression of FTD mutant tau led to increased trapping of Fyn in dendritic spines through increased tau self-assembly properties (Padmanabhan et al. 2019; Martínez-Mármol et al. 2023). Additionally, relative to wild-type tau, mutant tau has increased mislocalization to dendritic spines in mouse models and cultured neurons in a phosphorylation-dependent manner, where it dampens α-amino-3-hydroxy-5-methyl-4-isoxazolepropionic acid receptor (AMPAR)-mediated synaptic responses by reducing the presence of glutamate receptors (Hoover et al. 2010).

Links between tau and the complement cascade provide insight into the loss of synapses. Evidence in AD patient tissue and a P301S tau transgenic mouse model shows that C1q levels are increased at postsynapses and correlate with pathogenic tau (Dejanovic et al. 2018). Consistent with complement-mediated engulfment of synapses, synaptic markers were increased in microglial lysosomes (Dejanovic et al. 2018).

#### Mitochondrial dysfunction and oxidative stress

Mitochondria are particularly critical for the function and health of neurons, as they have a high metabolic requirement (Szabo et al. 2020). Associations with tau pathology and several aspects of mitochondrial homeostasis, such as transport and bioenergetics, have been reported in both animal models and patient tissue. Profiling of the tau interactome in cultured neurons has shown that wild-type tau can interact with mitochondrial proteins in a manner dysregulated by disease-associated mutations (Tracy et al. 2022). Tau-mediated toxicity may affect function through mitochondrial transport, as the distribution is altered in tauopathy model mice and AD patient tissue (Kopeikina et al. 2011). Impairments in mitochondrial respiration were also identified in mice expressing FTD-linked mutant tau (David et al. 2005). In line with a link between tau pathology and mitochondrial dysfunction, increased levels of reactive oxygen species can be detected in the AD hippocampus and correlates with the regional presence of tau oligomers in tauopathy model mice (Du et al. 2022). Thus, although there are associations of mitochondrial impairments with tau, a more precise understanding of the mechanistic basis as well as the contribution of this to overall neuronal dysfunction and death in disease is still needed.

#### RNA dysregulation

Another potential mechanism of tau-induced toxicity is the disruption of RNA homeostasis. Tau can bind RNA, RNA can serve as a cofactor for tau fibrillization, and tau aggregates contain RNA and RBPs (Lester and Parker 2024). For example, resident nuclear proteins involved in RNA processing such as U1 snRNP-associated, hnRNP, SR, and splicing speckle proteins have been shown to be sequestered in tau aggregates, which may disrupt their normal roles in splicing (Bai et al. 2013; Drummond et al. 2020; Lester et al. 2021). Consistent with this mechanism, RNA sequencing analysis of postmortem samples from AD patients shows dysregulated splicing (Apicco et al. 2019). Additionally, analysis of mRNA splicing changes in cultured cells with tau aggregates showed splicing defects with predominantly retained intron events (Lester et al. 2021). Furthermore, meta-analysis of tau interactome studies across human tissues and animal models shows consistent associations with RBPs, some of which preferentially interact with phosphorylated tau species but are not present in late stage tau aggregates such as neurofibrillary tangles, suggesting that they may be earlier mediators in the disease process (Kavanagh et al. 2022).

#### Nuclear cytoplasmic barrier

Potentially related to the mislocalization of nuclear factors, there is also evidence that pathogenic tau leads to disruptions in the nuclear cytoplasmic barrier. Tau can interact with the nucleoporin Nup98, which is mislocalized in neurons containing somatic phosphorylated tau in AD and primary tauopathies (Eftekharzadeh et al. 2018; Dickson et al. 2022). This is associated with disruption in the integrity of the nuclear pore complex (Eftekharzadeh et al. 2018). Tau has also been linked to the disruption of the nuclear membrane in cell models and FTD patient samples (Paonessa et al. 2019; Prissette et al. 2022).

Critically, although several cellular pathways are dysfunctional at end-stage disease, it is difficult to attribute causality in earlier stages of pathology. Much of the current insight comes from modulation of cellular pathways in mouse models and evaluation of effects on pathology. Overall, although we have some understanding of the mechanisms mediating tau-driven neurotoxicity, further work is required to understand the timing and contributions of various cellular pathways to overall pathology as well as the precise forms of tau eliciting deleterious effects. Important considerations as these questions are pursued are the aging-related impairments in cellular machinery that may not be effectively modeled in mice, whether distinct neurons are differentially vulnerable to tau-mediated toxic events, and what extent of tau pathology leads to either neuronal dysfunction or death. Ultimately, understanding the mechanistic basis of pathways downstream from pathogenic tau will be fundamental for evaluating the efficacy of tau-directed therapies and uncovering novel targets for the treatment of tauopathies.

## Tau targeted therapeutic outlooks

**Figure 7.**
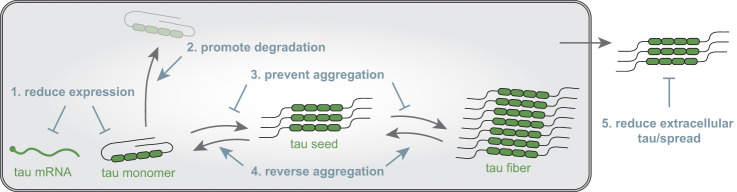
Tau targeted therapeutic approaches. Current therapeutic approaches targeting tau aim to reduce tau levels, promote degradation, prevent or reverse aggregation, or reduce extracellular levels of tau, thereby targeting spread.

Due to the promising nature of tau as a therapeutic target in preclinical findings, the tau therapeutic frontier is rapidly expanding. Tau targeted therapies in development have mainly focused on reducing the spread, aggregation, or levels of intracellular or extracellular tau (Fig. 7). There are currently no approved therapies directly targeting tau, and while many strategies are in preclinical stages or in clinical trials, to date it remains to be demonstrated that the desired modulation of tau in patients will lead to clinically meaningful improvements. However, this benchmark will likely be evaluated in the near future for several candidate approaches. Therapeutic strategies designed to modulate tau post-translational modifications such as kinase inhibition are not discussed here but are reviewed thoroughly elsewhere (Wang et al. 2021b). Here, we focus on therapeutic approaches aimed at reducing tau levels and the clearance or prevention of tau aggregates, including those being evaluated in clinical trials and novel approaches in preclinical stages.

### Antibody therapies

The largest number of tau-directed therapies currently being tested in clinical trials are classified as immunotherapies (Cummings et al. 2023). Several of these candidates rely on passive immunization with monoclonal anti-tau antibodies that differ in the region, post-translational modification, or conformation/oligomeric form of tau that is recognized. Highlighting the potential importance of the epitope, several major clinical trials of monoclonal antibodies targeting the N-terminal region of tau have failed to show benefit (Imbimbo et al. 2023). Thus, efforts have moved to produce antibodies against other regions of tau (in particular the MTBR critical for aggregation), which are actively being tested in clinical trials (Imbimbo et al. 2023). Another antibody-based approach being pursued is active vaccination against tau. Two candidates using synthetic peptides derived from fragments of the MTBR of tau have progressed to phase II clinical trials (Theunis et al. 2013; Novak et al. 2021; Cullen et al. 2024).

A key limitation applicable to most antibody approaches is that they target extracellular tau and thus are limited to acting at the level of tau spread rather than intracellularly where tau is mostly present or on other modes of spread where tau is shielded, such as in extracellular vesicles or tunneling nanotubes. Furthermore, there is a broad range of tau species due to alternatively spliced isoforms, numerous post-translational modifications, and the diversity of solved tau fibril structures. Thus, identification of an effective epitope is nontrivial and could differ across disease states.

### Therapeutics preventing or reversing tau aggregation

Several strategies to inhibit tau aggregation have also been developed. Methylene blue was shown to counter tau aggregation and hippocampal acetylcholine levels in preclinical studies but failed in phase II (Baddeley et al. 2015). A derivative hydromethylthionine mesylate has progressed to phase III trials where it failed to show clinical benefit (Gauthier et al. 2016), though additional trials have continued (Wischik et al. 2022). Many additional compounds have been identified as tau aggregation inhibitors through in vitro studies (for a more detailed review, see Wang et al. 2021b), but to date none have proven efficacious in clinical trials. In general, such compounds have many molecular targets and thus may lack sufficient specificity for tau in an in vivo setting.

In addition to small molecules, alternative approaches are in preclinical development that act at the level of tau aggregation. Novel peptide drugs have been designed that can bind to tau fibrils, preventing the formation of or even fragmenting existing fibrils in cellular and animal models (Aillaud and Funke 2023; Hou et al. 2024). Targeting of a ubiquitin ligase RING domain from tripartite motif-containing protein 21 (TRIM21) to tau aggregates through fusion with tau or a tau nanobody reverses and degrades aggregates, reducing pathology in tau transgenic mouse models (Benn et al. 2024; Miller et al. 2024).

### Strategies to reduce tau levels

Another approach to tackle tau-mediated toxicity is the general reduction of tau levels. Based on animal studies, the knockout of tau leads to a mild, late phenotype, suggesting that this strategy could be clinically feasible (Dawson et al. 2001; Morris et al. 2013).

One approach under development is an antisense oligonucleotide (ASO) that targets tau mRNA for degradation, thereby reducing protein levels. This ASO was effective at reducing pre-existing tau pathology in tau transgenic mice (DeVos et al. 2017). More recently, in a phase I trial, intrathecal delivery of the tau targeting ASO was well tolerated and reduced phosphorylated and total tau in the cerebral spinal fluid by ∼50% (Mummery et al. 2023). Promisingly, ASO-treated patients with mild AD showed reduced NFTs relative to placebo at 25 and 100 weeks of treatment, as measured through tau PET (Edwards et al. 2023). Currently, a randomized, double-blind, placebo-controlled phase II study is under way to evaluate efficacy (NCT05399888), which will provide insight into whether reducing the NFT burden has clinical benefit.

Additional strategies aim to harness the innate degradative pathways of cells for the targeted reduction of tau. This includes proteolysis targeting chimera (PROTAC) approaches that link tau and ubiquitin ligases through small molecules to enhance degradation (Silva et al. 2019; Wang et al. 2021c). Similarly, multifunctional peptides containing tau and E3 ligase binding elements have been designed and tested in cellular models (Chu et al. 2016; Lu et al. 2018). Furthermore, tau intrabodies have been developed that can be conjugated to ubiquitin, allowing directed turnover via the proteasome or lysosome (Gallardo et al. 2019). In another strategy, a tau PET probe was converted to bring together tau and a substrate receptor for the cullin4A–RING E3 ubiquitin ligase (CRL4), which can induce proteasomal degradation (Silva et al. 2019).

### Gamma entrainment using sensory stimuli (GENUS)

An alternative noninvasive strategy involves the entrainment of 40 Hz gamma-frequency brain rhythms by visual, auditory, or tactile stimulation. A series of studies has reported corrective effects of 40 Hz stimulation in AD and tauopathy disease models and suggests that this involves enhanced glymphatic clearance of protein aggregates and alteration of microglial transcriptional status from inflammatory to neuroprotective programs (Iaccarino et al. 2016; Adaikkan et al. 2019; Martorell et al. 2019; Suk et al. 2023; Murdock et al. 2024). However, other studies report a lack of entrainment with visual flickering or increased pathology with optogenetic stimulation, suggesting that further investigation is necessary to determine the precise mechanism of action and potentially optimize the mode of stimulation and entrainment (Wilson et al. 2020; Soula et al. 2023). Nonetheless, early human trials suggest that GENUS is well tolerated and can entrain 40 Hz oscillations in healthy and mild AD patients (Suk et al. 2020; Chan et al. 2022). Further clinical trials for 40 Hz entrainment by sound, noninvasive light, and transcranial alternating current stimulation are under way that will evaluate potential for disease-modifying effects.

## Perspectives and future directions

Although the solving of tau structures from disease has provided great insight into tau-driven pathogenesis, one important issue that remains to be resolved is the specific molecular interactions within neurons that modulate the dynamics of tau folding and incorporation into fibrils. This is likely to be complex, with multiple metabolites, proteins, and RNAs impacting tau aggregation. In this context, understanding the distinct cellular conditions in vulnerable neurons that exhibit tau aggregation earliest in specific diseases will provide valuable insights into specific cofactors and cell states that drive de novo tau misfolding. Additionally, understanding the molecular basis for how neuroinflammation affects the process of tau fibrilization, spread, and toxicity is key for developing new targets for disease mitigation.

In addition to how tau is folding and aggregating in disease, how tau exerts gain-of-function toxicities in various cell types is a key incompletely understood area. Elucidating which pathways and cellular functions are affected early in disease is paramount for finding novel biomarkers, enabling earlier diagnosis and treatment of tauopathies. Additionally, specific toxicities may be therapeutically targeted in combination with tau-directed approaches in order to maximize clinical benefit. Excitingly, tau-based therapeutics are progressing through clinical trials that will likely soon provide an indication in patients as to whether modulating tau will be a fruitful therapeutic approach.
